# Chemical composition, functional properties, physico‐chemical properties, and techno‐functional characteristics of Satureja protein hydrolysate stabilized in a gelatin matrix

**DOI:** 10.1002/fsn3.4344

**Published:** 2024-08-20

**Authors:** Elham Obeidnejad, Gholamreza Kavoosi, Mohammad Jamal Saharkhiz, Sayed Mohammad Shafiee

**Affiliations:** ^1^ Department of Biotechnology, School of Agriculture Shiraz University Shiraz Iran; ^2^ Department of Horticultural Science, School of Agriculture Shiraz University Shiraz Iran; ^3^ Department of Clinical Biochemistry, School of Medicine Shiraz University of Medical Sciences Shiraz Iran

**Keywords:** physico‐chemical, protein hydrolysates, Satureja, techno‐functional

## Abstract

The current evaluation was conducted to investigate the chemical composition and functional properties of protein hydrolysates from different *Satureja* species. The major amino acids (mg/100 g protein) in the protein from different *Satureja* species were arginine (6.06–14.37), asparagine (1.39–17.06), glutamine (2.42–11.60), glutamic acid (6.34–9.32), beta‐aminoisobutyric acid (3.31–10.29), alanine (4.64–9.59), aspartic acid (5.73–9.54), proline (4.25–8.14), leucine (3.63–5.69), serine (2.64–4.83), lysine (1.89–4.56), glycine (0.96–5.27), valine (2.76–4.83), and phenylalanine (2.86–3.75). Protein hydrolysates from *Satureja* species exhibited comparable antioxidant activity (IC_50_ = 0.270–0.328 mg/mL), anti‐linoleic acid oxidation (IC_50_ = 0.231–0.301 mg/mL), anti‐lecithin oxidation (IC_50_ = 0.198–0.258 mg/mL), anti‐starch oxidation (IC_50_ = 0.201–0.277 mg/mL), anti‐amylase activity (IC_50_ = 0.314–0.337 mg/mL), and anti‐lipase activity (IC_50_ = 0.309–0.369 mg/mL). The physico‐chemical properties of gelatin dispersion were electrical conductivity (1197 microsiemens/cm), osmolarity (190 milliosmol/kg), surface tension (45 mN/m), particle size (179 nm), zeta potential (−48 mV), and viscosity (4.92 mPa.s). Techno‐functional characteristics of freeze‐dried gelatin particles were as follows: water content (7.68%), water solubility (84.57%), water swelling (151.33%), hygroscopicity (38.20%), hydrophobicity (6.50 μg/g), emulsification activity (68.54%), emulsification stability (57.86%), foam expansion activity (56.08%), foam stability (41.84%), oil‐holding capacity (1.96 g/g), and water‐holding capacity (4.45 g/g). Protein hydrolysate causes minor changes in the physico‐chemical and techno‐functional properties of gelatin. The current investigation introduces *Satureja* protein hydrolysate as a possible functional and techno‐functional ingredient for bioactive food products for treating diabetes and oxidative stress and as a natural preservative for the food industry.

## Graphical abstract

Satureja had excellent essential/nonessential index and protein nutritional quality. Satureja protein hydrolysate exhibits excellent antioxidant capacity. Satureja protein hydrolysate inhibits amylase and lipase activities. Satureja protein is introduced as techno‐functional ingredient for bioactive food products.

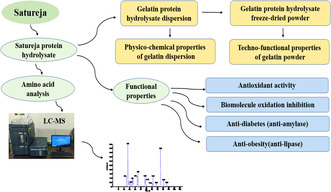

## 1 INTRODUCTION

The human population is increasing annually; however, as the population grows and the total land area remains constant, traditional animal husbandry will not satisfy the food needs of humans in the future. As key food components, proteins provide energy for the body's growth, development, and reproduction. Some examples of emergent and sustainable food sources include animals (beef, chicken, pork, meat, fish, and eggs), vegetables (grains, legumes, soybeans, wheat, chia, moringa, beans, and lentils), edible insects, and microorganisms (fungi and microalgae; Liu et al., 2024). Animal protein is susceptible to various environmental factors throughout the production process, including feed safety, public health hazards, environmental pollution, disease transmission, and other factors. Moreover, excessive consumption of meat can lead to cardiovascular disease and its subtypes, diabetes, and other diseases (Day et al., 2022). Many people are choosing to consume plant proteins instead of animal proteins. The utilization of plant‐derived proteins from herbal food by‐products is becoming increasingly important as the requirement for new protein sources develops concurrently with the global development of humanity. In this context, the use of proteins in the food industry is determined by protein properties, such as water solubility, water and oil storage ability, foaming properties, heat sensitivity, emulsifying properties, stability, and digestibility under acidic conditions (Nirmal et al., 2024). However, the native proteins generally have low solubility, low activity, and sometimes higher allergenicity. Partial hydrolysis of proteins by mild acid hydrolysis, chemical hydrolysis, enzymatic hydrolysis, and fermentation releases functional amino acids and generates bioactive peptides with several biological activities with higher solubility, higher activity, and lower allergenicity (Singh et al., 2023).

Bioactive peptides have become an emerging field of interest in the scientific community, along with the food, pharmaceutical, and cosmetics industries. The consumption of bioactive peptides may play a vital role in health through their broad spectrum of bioactivity, such as antioxidant, antihypertensive, antimicrobial, anti‐inflammatory, immunomodulatory, and anti‐proliferative activities. In addition, bioactive peptides can be used as food preservatives due to their antimicrobial and antioxidant activities (Bhandari et al., 2020). Some factors limit their nutraceutical and commercial applications, including easy chemical degradation, food matrix interaction, low water solubility, hygroscopicity, and potential bitter taste. The encapsulation of bioactive peptides in different materials can help overcome these challenges. The encapsulation of bioactive peptides increases their bioactivity, improves their stability and sensory properties, increases solubility, and decreases hygroscopicity (Aguilar‐Toalá et al., 2022). Gelatin is a natural polymer with numerous advantageous properties (low cost, biodegradability, biocompatibility, and availability of exposed chemical groups), that is widely used as a biomaterial to create microparticles in the biomedical field. It may be used in a range of synthesis processes, such as electrospray, desolvation, coacervation, nanoprecipitation, emulsion, and spray drying, to carry a variety of substances, due to its simplicity in production, accessibility, biocompatibility, minimal immunogenicity, and suitable biodegradability (Milano et al., 2023).

*Satureja*, or savory, is an aromatic plant that is commonly used as a culinary herb with significant therapeutic benefits. The major utility of *Satureja* is in improving the taste and/or smell of foods and being used as a remedy to cure indigestion, nausea, muscle cramps and pains, diarrhea, and infectious ailments (Sefidkon & Emami‐Bistgani, 2021). Recent phytochemical analyses have found the presence of carvacrol, monoterpene hydrocarbons, and flavonoids such as apigenin, luteolin, diosmetin, naringenin, taxifolin, aromadendrin, eriodictyol, and phenolic acids such as labiatic acid, and thymol in the aerial parts of *Satureja* (Babajafari et al., 2015). Moreover, carvacrol, cymene, terpinene, pinene, myrcene, thymol, linalool, and caryophyllene are the primary ingredients of *Satureja* (Maccelli et al., 2019). The observed antifungal, antioxidant, and anti‐bacterial properties suggest that *Satureja* could be a standard component within the nourishment sector to prevent food pollution, deterioration, and oxidation during processing (Jafari et al., 2018). *Satureja* also has the potential to regulate plasma lipids (Acimovic et al., 2021), decrease blood glucose (Acimovic et al., 2021), and inhibit lipid peroxidation (Jokanovj et al., 2020). There is also the argument of the pharmacological evidence for reducing inflammatory illnesses and managing lipid (hyperlipidemia) and sugar (hyperglycemia) in the blood using *Satureja* based on traditional utility (Saeidnia et al., 2016).

To the best of our knowledge, the amino acid/protein nutritional quality of *Satureja* and the amylase and lipase inhibitory capacities of *Satureja* protein hydrolysate have been less explored. Therefore, this study was conducted to examine the amino acid composition, nutritional value, antioxidant, anti‐biomolecule oxidation, anti‐amylase, and anti‐lipase activities of *Satureja* protein hydrolysate. Furthermore, protein hydrolysate was encapsulated in a gelatin dispersion. The physico‐chemical, and techno‐functional characteristics of gelatin dispersion and freeze‐dried gelatin particles were investigated. This is the first study that presents such a broad panel of chemical composition (proteinogenic amino acids and non‐proteinogenic amino acids), nutritional quality, and functional properties of protein hydrolysate in *Satureja*.

## 2 MATERIALS AND METHODS

### 2.1 Herbal materials

*Satureja hortensis* L. leaves were taken from an experimentation field. Following the identification of plant species, the voucher specimens (herbarium no. 538F19) were added to the herbarium at Shiraz University. Aerial parts of *S. sahendica* Bornm (Herbarium number: PH‐1582) were prepared from Mazhin, Lorestan, and *S. rechingeri* Jamzad (Herbarium number: PH‐1348) were collected from wild‐growing plants at the full flowering stage in Dehloran, Ilam (western parts of Iran). *S. bakhtiarica* Bunge (Herbarium number MPH‐1577) was also collected from Semirom, Isfahan (central part of Iran). Additionally, *S. khuzistanica* Jamzad (Herbarium number MPH‐1588) was collected from Mazhin, Lorestan. *S. mutika* (Herbarium number MPH‐1356) was prepared from Dehloran, Ilam (western districts of Iran) in the whole flowering level (Taban et al., 2021). Professor Ahmad Reza Khosravi (Faculty of Science, Shiraz University, Shiraz, Iran), as an experienced botanical taxonomist, taxonomically recognized and confirmed this plant. The plant material was dried in the shade and converted into powder in a household mixer for future utilities.

### 2.2 Protein hydrolyzing and profiling

Before the protein extraction process, fat and essential oil were removed by mixing *Satureja* powder with hexane (1:5 w/v) and stirring the mixture for 1 day at room temperature using a shaker at 150 rpm. The hexane phase (lipid and essential oil) was separated, and the defatted materials were dried. For protein extraction, defatted materials were mixed with a 300 mM NaCl solution (1:5 w/v). For complete solubilization of proteins and amino acids, the pH was adjusted to 9–10. The supernatant was separated by centrifugation at 3000 × *g* for 10 min. Eventually, the pH of the separated supernatant was adjusted to 7.0. The resulting protein suspension was stored in a refrigerator (4°C) until the experiments were done (Hashemirad et al., 2024).

To extract amino acids, the protein materials (1.0 g) were mixed with 10 mL of 6 M HCl and incubated at 100°C for 24 h. The undigested components were isolated by centrifugation at 3000 × *g* for 10 min. The obtained amino acid suspension was freeze‐dried and kept in a refrigerator until experiments. For amino acid profiling, the protein hydrolysate (10 mg/mL) was dissolved in a NaCl solution (0.30%). The chemical composition of protein hydrolysate was analyzed with liquid chromatography–tandem mass spectrometry (LC–MS/MS) (Agilent mass spectrometer, G1313) through an amino acid analyzer column with a 0.01 mL injection volume according to the previously published study (Heydari‐Koochi et al., 2023).

### 2.3 Protein nutritional parameters

According to the previous analysis (Heydari‐Koochi et al., 2023), the protein quality was evaluated by measuring the protein efficiency ratio, biological value, and the total profile of essential, nonessential, aromatic, hydrophobic, sulfur, flavor, bitter, and sweet amino acids (Table S1).

### 2.4 Antioxidant activity of *Satureja* protein hydrolysate

The partial hydrolysis of *Satureja* protein was obtained by mixing *Satureja* protein (1.0 g) with 10 mL of 2 M HCl and incubating at 80°C for 2 h. The undigested components were isolated by centrifugation at 3000 *g* for 10 min. The protein hydrolysate was freeze‐dried and kept in a refrigerator until experiments. SDS‐PAGE analysis revealed degradation of protein and production of small and heterogenous peptides (3–8 amino acids; Figure S1). The partial protein hydrolysate was diluted with 1.0% NaCl to a concentration of 10 mg/mL. According to the literature (Heydari‐Koochi et al., 2022), the antioxidant capacity was assessed by mixing the protein hydrolysate with ABTS stock solution (Sigma‐Aldrich, St Louis, MO, USA) and recording the light absorbance value at a wavelength of 734 nm. Trolox was used as a control in this analysis. The percentage of radical scavenging was determined according to the previous reports published in the field (Siahbalaei & Kavoosi, 2021).

### 2.5 Biomolecule oxidation inhibition

The effects of protein hydrolysate on biomolecule oxidation (starch, lecithin, and linoleic acid) were evaluated according to the procedures stated in the literature (Siahbalaei et al., 2020; Siahbalaei et al., 2021). Before the experiments, stock solutions of soluble starch (10 mg/mL), lecithin (10 mg/mL), and linoleic acid (10 mg/mL) were prepared, respectively. In the parallel experiments, the oxidant solution for producing hydroxyl radicals was prepared by CuSO_4_ (1.0 mM) plus H_2_O_2_ (5.0 mM). 1.0 mL of different concentrations of protein hydrolysate (0–300 μg/mL) were incubated with an equal value (1.0 mL) of biomolecule stock solution and incubated at 37°C for 1 h. Then, an equal volume (1.0 mL) of the oxidant reaction solution was added to the biomolecule‐protein hydrolysate mixture. The biomolecule‐protein hydrolysate‐oxidant solution was incubated at 37°C for 24 h (Trolox was used as the control). After this, the final absorbance of the biomolecule‐essential oil‐oxidant solution was recorded at 235 nm (for lipid oxidation) and 310 nm (for starch oxidation). The percent of biomolecule oxidation inhibition was calculated according to the previous reports (Siahbalaei & Kavoosi, 2021).

### 2.6 Amylase inhibition capacity

According to the literature (Heydari‐Koochi et al., 2022; Siahbalaei et al., 2021), 0.4 mL of protein hydrolysate was added to amylase (2.0 U/mL) and then placed at 37°C for 20 min to assess the α‐amylase (EC 3.2.1.1; Sigma‐Aldrich, St Louis, MO, USA) inhibition capacity. 0.25 mL of starch solution (10 mg/mL) was added to the mixture and incubated at 37°C for 30 min. Then, 0.1 mL of iodine reagent was added, and the absorbance was recorded at 580 nm. According to the previous reports, the concentration leading to 50% amylase inhibition (IC_50_) was calculated based on the change observed in light absorbance with inhibitor concentration (Siahbalaei & Kavoosi, 2021).

### 2.7 Lipase inhibition capacity

2.0 U/mL of lipase was mixed with 0.03 mL *Satureja* protein hydrolysate and 0.03 mL of orlistat, followed by a 30‐min incubation at 37°C to ascertain the lipase inhibition activity. P‐nitrophenyl butyrate solution (pNPB, 0.03 mL, 10 mg/mL; Sigma‐Aldrich) was then added to the mixture, and the light absorption was recorded at 405 nm for every 3 min. The IC_50_ of lipase inhibition was calculated based on the change in light absorbance with inhibitor concentration according to the previous studies (Siahbalaei & Kavoosi, 2021).

### 2.8 Preparation of the gelatin solution

Gelatin (type A from porcine skin, Sigma‐Aldrich) was dissolved in distilled water at 45°C in a 10% w/v ratio. To complete the solubilization of gelatin powder, the pH of the gelatin solution was adjusted to 9–10 by the addition of 0.5 mL ethanolamine (also as a cross‐linker) to the gelation solution (5.0% w/w based on polymer; Wu et al., 2022). Likewise, protein hydrolysate was dissolved at 50 mg/mL in NaCl (0.1%). 1.0, 2.0, and 3.0 mL of the protein hydrolysate were respectively added to the gelatin solution, and the final concentration of protein hydrolysate was found to be 0.50, 1.00, and 1.50 mg/mL of the gelatin solution. The alkaline gelatin solution was then mixed and placed at 50°C for 12 h. Glycerol (20% w/w), as a plasticizer, was also added to the alkaline gelatin solution. The alkaline gelatin solution was homogenized to complete the emulsification of the ingredients. The final volume was adjusted to 100 mL. The alkaline gelatin solution without protein hydrolysate was prepared in the same way (Siahbalaei et al., 2020).

### 2.9 Physico‐chemical properties of gelatin solution

The physico‐chemical and rheological characteristics of a diluted alkaline gelatin solution (2.0%), including conductivity, osmolarity, zeta‐potential, dynamic particle size, viscosity, and surface tension, were determined according to the practical approach at an ambient temperature according to previous research (Attarian et al., 2024).

### 2.10 Techno‐functional properties of gelatin particles

Before experiments, gelatin dispersions were powdered by freeze‐drying. The techno‐functional properties of powdered materials, including water content, water solubility, water activity, hygroscopicity, surface hydrophobicity, emulsification activity, emulsification stability, foam expansion activity, foam stability, oil‐holding capacity, and water‐holding capacity, were measured as previously described (Hashemirad et al., 2024).

### 2.11 Statistical analyses

All data are provided as mean ± SD. The one‐way analysis of variance (ANOVA) and Tukey's post‐hoc test were used to examine significant differences among samples using the SPSS software (SPSS, Abaus Concepts, Berkeley, CA). A *p*‐value of less than .05 (*p* < .05) was considered to be statistically significant.

## 3 RESULTS AND DISCUSSION

### 3.1 Chemical composition of amino acid

The major amino acids (mg/100 g protein) in the protein from different *Satureja* species were arginine (6.06–14.37), asparagine (1.39–17.06), glutamine (2.42–11.60), glutamic acid (6.34–9.32), beta‐aminoisobutyric acid (3.31–10.29), alanine (4.64–9.59), aspartic acid (5.73–9.54), proline (4.25–8.14), leucine (3.63–5.69), serine (2.64–4.83), lysine (1.89–4.56), glycine (0.96–5.27), valine (2.76–4.83), and phenylalanine (2.86–3.75; Table 1). The highest standard deviation between amino acids from Satureja species belongs to asparagine (6.24), followed by citrulline (4.36), glutamine (3.06), arginine (2.91), beta‐aminoisobutyric acid (2.58), alanine (2.11), glycine (1.51), aspartic acid (1.41), proline (1.34), glutamic acid (1.06), and lysine (1.02). The yield of protein hydrolysate was about 12–14 g per 100 g of dried powder. As far as our knowledge, there is no report on the amino acid composition of the Satureja. Limited work has been conducted on the elucidation of the amino acid composition and quality of other Lamiaceae families. However, these rare reports suggested that the Lamiaceae family (*Oliveria decumbens*, *Thymus kotschyanus*, *Trachyspermum ammi*, and *Zataria multiflora*) has a good balance of asparagine (0.12–3.67 g), arginine (0.42–1.31 g), beta‐aminoisobutyric acid (0.33–0.94 g), glutamine (0.20–0.54 g), aspartic acid (0.05–0.91 g), glutamic acid (0.07–0.49 g), proline (0.05–0.49 g), alanine (0.20–0.33 g), phenylalanine (0.04–0.32 g), valine (0.05–0.24 g), leucine (0.05–0.23 g), and serine (0.03–0.24 g) per 100 g protein that is, to some extent, similar to our results (Siahbalaei & Kavoosi, 2021). Amino acids have various protective effects, including osmoprotectant, cellular enzyme stabilization, reactive oxygen species scavenging, maintaining redox balance, regulators of energy balance, nutrition metabolism, gastrointestinal health, immunity, and intestinal health, and Satureja protein could be a promising applicant for these proposes (Nie et al., 2018).

**TABLE 1:** Amino acid composition (g/100 g protein) of *Satureja.*

Amino acid	*Satureja hortensis*	*S. rechingeri*	*S. sahendica*	*S. bakhtiarica*	*S. khuzistanica*	*S. mutika*	Mean	SD
Alanine	6.10 ± 0.35b	9.40 ± 0.54a	5.16 ± 0.30c	4.60 ± 0.27c	9.58 ± 0.55a	6.89 ± 0.45b	6.96	2.11
Allo‐isoleucine	0.005 ± 0.00a	0.010 ± 0.00a	0.007 ± 0.00a	0.01 ± 0.00a	0.01 ± 0.00a	0.01 ± 0.00a	0.01	0.00
Alpha‐aminobutyric acid	0.10 ± 0.01a	0.03 ± 0.00b	0.04 ± 0.00b	0.01 ± 0.00c	0.02 ± 0.00c	0.06 ± 0.02a	0.04	0.03
Arginine	6.07 ± 0.35d	9.19 ± 0.53c	11.13 ± 0.64b	12.24 ± 0.71b	14.37 ± 0.83a	8.80 ± 0.50c	10.30	2.91
Asparagine	10.95 ± 0.63b	4.48 ± 0.26c	17.06 ± 0.99a	1.39 ± 0.08d	1.67 ± 0.10d	10.83 ± 0.55b	7.73	6.24
Aspartic acid	9.54 ± 0.55a	6.47 ± 0.37c	6.48 ± 0.37c	5.95 ± 0.34c	5.72 ± 0.33c	7.50 ± 0.38b	6.94	1.41
Beta‐alanine	0.05 ± 0.00a	0.03 ± ±0.00a	0.04 ± 0.00a	0.02 ± 0.00a	0.05 ± 0.00a	0.04 ± 0.02a	0.04	0.01
Beta‐aminoisobutyric acid	3.30 ± 0.19d	8.15 ± ±0.47b	10.28 ± 0.60a	9.09 ± 0.53a	5.17 ± 0.30c	7.25 ± 0.40b	7.21	2.58
Citrulline	0.06 ± 0.00e	0.07 ± 0.00e	1.13 ± 0.07b	11.00 ± 0.64a	0.15 ± 0.01d	0.42 ± 0.25c	2.14	4.36
Cystine	1.07 ± 0.06a	1.33 ± 0.08a	1.10 ± 0.06a	1.19 ± 0.07a	0.38 ± 0.02b	1.17 ± 0.53a	1.04	0.34
Gamma‐aminobutyric acid	0.18 ± 0.01c	0.14 ± 0.01c	1.05 ± 0.06a	0.05 ± 0.00d	0.11 ± 0.01c	0.46 ± 9.23b	0.33	0.38
Glutamic acid	7.09 ± 0.41b	6.86 ± 0.40b	6.80 ± 0.39b	6.33 ± 0.37b	9.32 ± 0.54a	6.92 ± 0.40b	7.22	1.06
Glutamine	7.42 ± 0.43c	9.20 ± 0.53b	6.33 ± 0.37c	2.42 ± 0.14d	11.59 ± 0.67a	7.65 ± 0.48c	7.44	3.06
Glycine	4.29 ± 0.25b	4.27 ± 0.25b	0.96 ± 0.06d	5.26 ± 0.30a	4.34 ± 0.25b	3.17 ± 0.22c	3.72	1.51
Glycylproline	0.02 ± 0.00a	0.06 ± 0.00a	0.01 ± 0.00a	0.01 ± 0.00a	0.07 ± 0.00a	0.03 ± 0.01a	0.03	0.03
Histidine	2.02 ± 0.12a	1.76 ± 0.10a	1.26 ± 0.07b	2.06 ± 0.12a	1.75 ± 0.10a	1.68 ± 0.11a	1.76	0.29
Hydroxyproline	0.20 ± 0.0b	0.50 ± 0.03b	0.13 ± 0.01c	0.49 ± 0.03a	0.57 ± 0.03a	0.28 ± 0.02b	0.36	0.18
Isoleucine	2.35 ± 0.14a	1.74 ± 0.10b	2.43 ± 0.14a	2.29 ± 0.13a	1.48 ± 0.09b	2.17 ± 0.12a	2.08	0.38
Leucine	4.64 ± 0.27b	4.23 ± 0.24b	3.63 ± 0.21c	5.69 ± 0.33a	4.51 ± 0.26b	4.17 ± 0.25b	4.48	0.69
Lysine	4.28 ± 0.25a	4.27 ± 0.25a	1.89 ± 0.11b	4.56 ± 0.26	4.43 ± 0.26	3.48 ± 0.20	3.82	1.02
Methionine	0.72 ± 0.04a	0.69 ± 0.04a	0.84 ± 0.05a	0.71 ± 0.04a	0.79 ± 0.05a	0.75 ± 0.04a	0.75	0.05
Ornithine	2.41 ± 0.14a	0.23 ± 0.01d	0.16 ± 0.01c	0.40 ± 0.02c	0.27 ± 0.02d	0.94 ± 0.06b	0.74	0.87
Phenylalanine	4.41 ± 0.26a	3.36 ± 0.19a	3.05 ± 0.18a	3.74 ± 0.22a	2.85 ± 0.17b	3.61 ± 0.22a	3.51	0.56
Proline	5.22 ± 0.30c	8.14 ± 0.47a	6.69 ± 0.39b	4.20 ± 0.25d	6.25 ± 0.36b	6.69 ± 0.33b	6.21	1.34
Serine	4.82 ± 0.28a	4.66 ± 0.27a	2.64 ± 0.15c	3.70 ± 0.22b	4.80 ± 0.28a	4.04 ± 0.24a	4.13	0.85
Threonine	2.29 ± 0.13b	2.82 ± 0.16a	1.93 ± 0.11c	2.95 ± 0.17a	2.55 ± 0.15b	2.35 ± 0.17b	2.48	0.37
Tryptophan	2.62 ± 0.15a	2.22 ± 0.13a	2.10 ± 0.12a	2.60 ± 0.16a	2.15 ± 0.12a	2.32 ± 0.18a	2.35	0.25
Tyrosine	2.37 ± 0.14a	1.89 ± 0.11ab	1.59 ± 0.09b	2.35 ± 0.13a	1.60 ± 0.10b	1.95 ± 0.14b	1.96	0.32
Valine	4.82 ± 0.28a	3.07 ± 0.18b	3.46 ± 0.20b	3.555 ± 0.21b	2.760 ± 0.16c	3.79 ± 0.21b	3.58	0.71
Total	99.47 ± 5.76a	99.34 ± 5.75a	99.41 ± 5.75a	99.12 ± 5.74a	99.45 ± 5.76a	99.41 ± 5.00a	99.37	0.13
Nonessential amino acid	64.95 ± 3.76a	65.91 ± 3.81a	65.96 ± 3.82a	49.75 ± 2.88b	69.70 ± 4.04a	65.61 ± 4,15a	63.65	7.01
Essential amino acid	28.18 ± 1.63a	24.18 ± 1.40b	20.59 ± 1.19c	28.22 ± 1.63a	23.30 ± 1.35b	24.32 ± 1.50b	24.81	2.96
Ketogenic amino acid	20.69 ± 1.30a	17.72 ± 1.34b	14.70 ± 1.00c	21.29 ± 1.50a	17.10 ± 1.30b	17.70 ± 1.20b	18.20	2.44
Glucogenic amino acid	72.44 ± 2.70a	72.37 ± 3.00a	71.85 ± 2.85a	56.73 ± 2.30b	75.95 ± 3.40a	72.22 ± 3.50a	70.26	6.80
Branched‐chain amino acid	11.82 ± 0.85a	9.05 ± 0.70b	9.52 ± 0.60ab	11.54 ± 0.73a	8.76 ± 0.50b	10.13 ± 0.55a	10.13	1.29
Non‐protein amino acid	6.34 ± ±0.37d	9.24 ± 0.54c	12.86 ± 0.74b	21.15 ± 1.22a	6.45 ± 0.37d	9.48 ± 1.00c	10.91	5.54
Flavor amino acid	16.63 ± 0.96a	13.33 ± 0.77b	13.28 ± 0.77b	12.25 ± 0.71b	15.05 ± 0.87a	14.42 ± 0.84a	14.17	1.54
Aromatic amino acid	9.41 ± 0.54a	7.48 ± 0.43b	6.74 ± 0.39b	8.70 ± 0.51a	6.60 ± 0.39b	7.88 ± 0.42b	7.82	1.09
Sweet amino acid	22.72 ± 1.32b	29.31 ± 1.70a	17.38 ± 1.01c	20.80 ± 1.21b	27.50 ± 1.60a	23.14 ± 1.55b	23.50	4.37
Bitter amino acid	22.85 ± 1.32b	23.20 ± 1.34b	24.44 ± 1.41b	29.42 ± 1.70a	27.95 ± 1.62a	23.50 ± 1.50b	25.23	2.77
Sulfur amino acid	1.79 ± 0.10a	2.02 ± 0.12a	1.94 ± 0.11a	1.90 ± 0.11a	1.17 ± 0.07a	1.92 ± 0.08a	1.79	0.31
Hydrophobic amino acid	28.27 ± 1.64a	30.65 ± 1.77a	25.27 ± 1.46b	24.88 ± 1.44b	28.24 ± 1.63a	28.06 ± 1.70a	27.56	2.15
Basic amino acid	12.38 ± 0.72c	15.23 ± 0.88b	14.28 ± 0.83b	18.87 ± 1.09a	20.56 ± 1.19a	13.96 ± 0.80c	15.88	3.15
Acidic amino acid	16.63 ± 0.96a	13.34 ± 0.77b	13.28 ± 0.77b	12.29 ± 0.71b	15.05 ± 0.87a	14.42 ± 0.88a	14.17	1.54
Protein efficiency ratio‐1	1.19 ± 0.07b	0.86 ± 0.05b	0.65 ± 0.04c	1.71 ± 0.10a	1.08 ± 0.06b	0.90 ± 0.05b	1.07	0.37
Protein efficiency ratio‐2	1.39 ± 0.08b	1.25 ± 0.07b	1.01 ± 0.06c	1.87 ± 0.11a	1.41 ± 0.08b	1.22 ± 0.07b	1.36	0.29
Protein efficiency ratio‐3	0.35 ± 0.03c	0.41 ± 0.02c	0.17 ± 0.01d	1.23 ± 0.07a	0.89 ± 0.05b	0.31 ± 0.02c	0.56	0.41
EAA/NEAA index	0.43 ± 0.035b	0.37 ± 0.024c	0.31 ± 0.023c	0.57 ± 0.04a	0.33 ± 0.03c	0.37 ± 0.03c	0.40	0.09

*Note*: The values are expressed as means ± SD for three replicates. Mean values with different letters within a row are significantly different (*p* < .05).

### 3.2 Nutritional quality of protein hydrolysate

The primary amino acids of protein hydrolysate from different *Satureja* species are glucogenic (56.73–75.95 mg), hydrophobic (24.88–30.65 mg), bitter (22.85–29.43 mg), sweet (19.39–29.31 mg), ketogenic (14.70–21.29 mg), basic (12.38–20.56 mg), flavor (12.29–15.05 mg), acidic (12.29–16.63 mg), branched‐chain (8.76–11.82 mg), aromatic (6.67–9.41 mg), and sulfur (1.17–2.02 mg) amino acids per 100 g protein (Table 1). Protein hydrolysates are mainly composed of nonessential amino acids (49.78–68.73 mg), essential amino acids (20.59–28.24 mg), and non‐protein amino acids (6.35–21.12 mg) per 100 g of protein. Given the protein nutritional quality, protein hydrolysate had reasonable amounts of protein efficiency ratio‐2 (1.01–1.87), protein efficiency ratio‐1 (0.66–1.71), protein efficiency ratio‐3 (0.18–1.23), and essential/nonessential amino acid index (0.310–0.567; Table 1). As far as our knowledge, there is no report on the protein nutritional quality of the *Satureja*. Limited work has been conducted on the elucidation of the amino acid quality of other Lamiaceae families (*O. decumbens*, *T. kotschyanus*, *T. ammi*, and *Z. multiflora*), suggesting that the results of this study are to some extent similar to the previous results (Siahbalaei & Kavoosi, 2021). Due to its high essential amino acid content and biological value, *Satureja* has better protein nutritional qualities than common plants. Regarding the linkage between proline‐leucine (PER1), tyrosine‐leucine (PER2), and leucine–methionine–histidine–tyrosine (PER3), the *Satureja* protein has a higher nutritional value compared to other plant proteins (Kowalczewski et al., 2019). Sulfur‐containing amino acids participate in the production of cellular antioxidants such as n‐acetylcysteine and glutathione to remove harmful heavy metals (Colovic et al., 2018). Hydrophobic and aromatic amino acids have cellular antioxidant activity, reducing oxidative stress biomarkers, increasing the activity of various antioxidant enzymes, and regulating the content of antioxidant molecules (Aguilar‐Toalá & Liceaga, 2021). Flavor amino acids have a positive effect on regulating gastrointestinal function and can be used to treat gastric diseases (Zhang et al., 2020). Ketogenic amino acids have been reported to induce autophagy and alleviate the course of the disease, and they are anti‐tumor, anti‐cancer, anti‐angiogenesis, and anti‐inflammatory beneficial (Srivastava et al., 2022). Supplementing with branched‐chain amino acids before and after exercise could reduce muscle damage induced by long‐term exercise (Holeček, 2018).

### 3.3 Antioxidant activity

Protein hydrolysates from *Satureja* species with different constituents exhibited comparable antioxidant activity (IC_50_ = 0.270–0.328 mg/mL), anti‐linoleic acid oxidation (IC_50_ = 0.231–0.301 mg/mL), anti‐lecithin oxidation (IC_50_ = 0.198–0.258 mg/mL), and anti‐starch oxidation (IC_50_ = 0.201–0.277 mg/mL). Protein hydrolysate inhibited biomolecule (linoleic acid, lecithin, and starch) oxidation induced by hydroxyl radicals in a similar manner (Table 2). The antioxidant activity of essential oils from *Satureja* has been extensively studied. However, the antioxidant activity of the protein hydrolysate from *Satureja* was not explored. The results of this study are to some extent similar to the results of the anti‐glucose oxidation (IC_50_ = 0.235–0.280 mg/mL), anti‐lipid oxidation (IC_50_ = 0.190–0.270 mg/mL), and anti‐protein oxidation (IC_50_ = 0.210–0.320 μg/mL) of protein hydrolysate obtained from *O. decumbens*, *T. kotschyanus*, *T. ammi*, and *Z. multiflora* (Siahbalaei & Kavoosi, 2021). Hydroxyl radicals produced by the hydroxyl radical‐producing system (CuSO_4_ + H_2_O_2_) oxidized glucose, lipids, and proteins, leading to significant alterations in cell signaling and damage (Chitra et al., 2020). Protein hydrolysates as antioxidants and hydroxyl radical scavengers inhibit glucose, lipid, and protein oxidation. The effectiveness of protein hydrolysate in antioxidant activity and scavenging free radicals could be attributed to the antioxidant amino acids, such as aromatic amino acids (tyrosine, phenylalanine, and tryptophan), hydrophobic amino acids (proline, cysteine, methionine, and phenylalanine), basic amino acids (arginine, lysine, and histidine), acidic amino acids (glutamate and aspartate), and sulfur amino acid (cysteine and methionine) due to the ability to donate protons or electrons to convert free radicals into stable forms (Huang et al., 2024). Therefore, the scavenging of free radicals and antioxidant activity of *Satureja* protein hydrolysate may be related to the enhanced release of hydrophobic/aromatic/acidic/sulfur amino acids after partial protein hydrolysis.

**TABLE 2:** Total antioxidant activity (IC_50_, μg/mL), linoleic oxidation inhibition, lecithin oxidation inhibition, starch oxidation inhibition, anti‐amylase capacity, and anti‐lipase capacity of Satureja protein hydrolysate.

Samples	ABTS radical	Starch oxidation	Linoleic oxidation	Lecithin oxidation	Amylase inhibition	Lipase inhibition
Control	160 ± 5.5e	150 ± 4.7e	115 ± 3.5d	120 ± 4.0e	155 ± 4.5c	205 ± 6.3d
*SSatureja bakhtiarica*	294 ± 9.0bc	214 ± 6.5d	266 ± 8.0b	230 ± 7.0bc	341 ± 10a	321 ± 9.5bc
*S. hortensis*	310 ± 10ab	201 ± 6.0d	231 ± 7.0c	234 ± 7.4b	323 ± 11ab	352 ± 11ab
*S. khuzistanica*	270 ± 8.0d	230 ± 7.0c	293 ± 9.5a	218 ± 6.5c	328 ± 9.0a	309 ± 9.0c
*S. mutika*	319 ± 11a	253 ± 8.0b	244 ± 7.0c	198 ± 6.0d	333 ± 8.5a	362 ± 8.6a
*S. rechingeri*	282 ± 8.7 cd	277 ± 10a	301 ± 10a	238 ± 7.5b	314 ± 9.5b	313 ± 10c
*S. sahendica*	328 ± 10a	250 ± 8.5b	280 ± 9.0ab	258 ± 8.0a	337 ± 10a	369 ± 9.7a

*Note*: Total antioxidant activity, linoleic oxidation inhibition, lecithin oxidation inhibition, starch oxidation inhibition, protein oxidation inhibition, anti‐amylase capacity, and anti‐lipase capacity were expressed as concentrations needed for 50% (IC_50_) oxidant scavenging or enzyme inhibition. The values are expressed as means ± SD for three experimental replicates (*n* = 3). The Tukey test's mean values with different letters within a row differ significantly (*p* < .05).

### 3.4 Anti‐amylase

Protein hydrolysates from Satureja species with different constituents exhibited comparable anti‐amylase activity (IC50 = 0.313–0.369 mg/mL; Table 2). The results of this study are, to some extent, similar to the results of the anti‐amylase activity (IC_50_ = 0.300–0.480 mg/mL) of protein hydrolysate obtained from *O. decumbens*, *T. kotschyanus*, *T. ammi*, and *Z. multiflora* (Siahbalaei & Kavoosi, 2021). Recent investigations established the potential inhibitory effects of bioactive peptides (3–8 amino acids) from germinated soybean on glucosidase (IC_50_ = 2.90 mg/mL) and amylase (IC_50_ = 1.7 mg/mL) inhibition (González‐Montoya et al., 2018). Nonhydrolyzed camel whey protein weakly inhibits amylase (IC_50_ = 6.42 mg/mL) and glucosidase (27 mg/mL), but hydrolyzed camel whey protein strongly inhibits amylase (IC_50_ = 1.33 mg/mL) and glucosidase (IC_50_ = 1.12 mg/mL; Baba et al., 2021). The quinoa protein hydrolysate showed α‐amylase inhibitory (IC_50_ = 2.5–4.0 mg/mL) activity (Zhou et al., 2023). The anti‐amylase activity of peptides is very different and may be related to the synergistic effects of hydrophobic, cyclic, branched, and aromatic amino acids. Enzyme inhibitory activity seems to be marked for peptides containing arginine, serine, tyrosine, threonine, or lysine at the N‐terminus and methionine, proline, or alanine residues near the C‐terminus (Di Stefano et al., 2018). A role for hydrophobic amino acids and hydrophobic interactions in amylase inhibition has been confirmed (Sarabandi et al., 2023). Noncovalent interactions between the enzyme's active site and the peptide are critical for these inhibitions. Amylase inhibition may be mediated by any *Satureja* amino acids, including branched‐chain/cyclic chain/aromatic amino acids in protein hydrolysate (Fallah et al., 2022). Small amino acids may reside in the amylase active site or act as carbohydrate blockers by limiting carbohydrate breakdown. Large amino acids can bind to an allosteric region, changing the enzyme conformation and slowing the breakdown of polysaccharides (Navarro‐Leyva et al., 2023). The structure of aromatic/branched/cyclic amino acids is all related to their ability to block enzymes. The binding of these bioactive compounds to the active and allosteric sites of enzymes results in conformational changes and enzyme inhibition (Morais et al., 2020).

### 3.5 Anti‐lipase activity

Protein hydrolysates from *Satureja* species with different constituents exhibited comparable anti‐lipase activity (IC_50_ = 0.314–0.341 mg/mL; Table 2). The previous results suggested that seabuckthorn seed protein hydrolysate inhibited lipase activity by 35% at 250 mg/100 g protein. This anti‐lipase activity of amino acids may be related to the synergistic effects of hydrophobic amino acids with branched‐chain, cyclic‐chain, and aromatic‐chain amino acids (Xiang et al., 2020). The previous results suggested that the hydrolyzed oat proteins and peptides inhibit lipase (IC_50_ = 85.4 μM) and α‐amylase (IC_50_ = 37.5 μM; Esfandi et al., 2022). Furthermore, yellow‐field pea protein hydrolysates inhibit lipase (IC_50_ = 4.2 mg/mL; Awosika & Aluko, 2019). Pearl millet protein hydrolysates exhibit lipase inhibitory activity (IC_50_ = 3.46–3.60 μg/mL; Mudgil et al., 2023). The effective anti‐lipase activity of proteins hydrolysate is very diverse. It could be attributed to the antioxidant amino acids, such as aromatic amino acids (tyrosine, phenylalanine, and tryptophan), hydrophobic amino acids (proline, cysteine, methionine, and phenylalanine), basic amino acids (arginine, lysine, and histidine), acidic amino acids (glutamate and aspartate), and sulfur amino acids (cysteine and methionine) due to their ability to donate protons or electrons to convert free radicals into stable forms (Zhao et al., 2024). By limiting the breakdown of triglycerides, small molecules can reside in the triglyceride active site of lipase or act as a triglyceride blocker. Large molecules can bind to allosteric regions in lipase, causing a conformational change, lowering lipase affinity for triglycerides, and slowing triglyceride breakdown (Ashaolu et al., 2024).

### 3.6 Physio‐chemical properties of gelatin–protein hydrolysate dispersion

The physio‐chemical behaviors of gelatin solution were electrical conductivity (1197 microsiemens/cm), osmolarity (190 milliosmol/kg), surface tension (4 mN/m), zeta potential (−48 mV), particle size (179 nm), particle size distribution (0.51), shear stress (246 mPa), and viscosity (4.92 mPa.s; Table 3). The physio‐chemical behaviors of the gelatin solution in this research are similar to the previous results; however, there are some significant differences due to the gelatin source and the mode of gelatin preparation (De Maeseneer et al., 2024). Adding protein hydrolysate to gelation dispersion causes minor changes in pH (decrease), electrical conductivity (increase), osmolarity (increase), surface tension (increase), zeta potential (increase), particle size (increase), shear stress (decrease), and viscosity (decrease) of the gelatin solution (Table 3). Our experimental results are similar to the results of *Zataria multiflora* essential oils encapsulated in gelatin‐pectin particles (Siahbalaei et al., 2020) and *Satureja bakhtiarica* incorporated in gelatin particles (Obeidnejad et al., 2024). Encapsulation of bitter peptides in water‐in‐oil high internal phase emulsions (Gao, Wu, et al., 2022) and in diphasic gel double emulsions (Gao, Li, et al., 2022) improves the physico‐chemical properties and physical stability of peptides. The viscoelasticity of a gelatin solution depends on the shear rate (flow rate), particle concentration, particle shape (spherical, nonspherical, ellipsoid, regular, irregular, star‐shaped), particle size distribution (polydisperse or monodisperse), particle surface charge (zeta‐potential), electrical conductivity, surface tension, and osmolarity. The changes in physico‐chemical properties of gelatin solution may be attributed to the hydrophilic nature and negative charge of protein hydrolysate. The negative surface charge, small particle size, and low polydispersity decrease the surface and friction force between particles, leading to viscosity reduction (Shakeel et al., 2019). Additionally, protein solubility, hydrodynamic characteristics, hydrophobicity, and microstructure have substantial roles in rheological properties. The viscosity of proteins depends on the interactions between proteins and water and between hydrated proteins with each other. Protein hydrolysates increase the water‐binding capacity of gelatin and reduce the frictional forces between particles. Subsequently, the contact between the particles is reduced and the viscosity decreases (Shakeel et al., 2019). Physico‐chemical behaviors (including zeta‐potential, particle size, and particle size distribution) and visual observations suggested the good dispersion of protein hydrolysate in the gelatin network and the compatibility of protein hydrolysate with the gelatin matrix. The good dispersion of the peptides in the gelatin increases peptide stability, reduces particle aggregation, reduces phase separation, and increases the bioactivity of peptides (Ghorani et al., 2020).

**TABLE 3:** Physico‐chemical and techno‐functional properties of gelatin incorporated with Satureja protein hydrolysate.

	Gelatin	Gelatin‐ protein hydrolysate 1	Gelatin‐ protein hydrolysate 2	Gelatin‐ protein hydrolysate 3
Physico‐chemical properties				
pH of solution	10 ± 0.25a	9.9 ± 0.24a	9.7 ± 0.23ab	9.3 ± 0.20b
Conductivity (μS/cm)	1197 ± 14b	1200 ± 15b	1235 ± 18a	1250 ± 17a
Surface tension (mN/m)	45 ± 1.40b	46 ± 1.50ab	47 ± 1.70ab	49 ± 1.80a
Osmolarity (mOsmol/kg)	190 ± 4.4c	196 ± 4.5c	210 ± 5.0b	225 ± 5.3a
Electrophoretic mobility (−mm^2^/Vs)	0.039 ± 0.001b	0.041 ± 0.001b	0.044 ± 0.001a	0.046 ± 0.001a
Zeta‐potential (‐mV)	48 ± 1.25d	52 ± 1.30c	55 ± 1.33b	60 ± 1.45a
Particle size (nm)	179 ± 4.0d	195 ± 4.5c	240 ± 4.7b	270 ± 6.4a
Polydispersity	0.518 ± 0.01d	0.816 ± 0.02c	0.936 ± 0.02b	0.986 ± 0.02a
Shear stress (mPa)	246 ± 6.0a	232 ± 5.0b	211 ± 4.7c	186 ± 4.0d
Viscosity (mPa.s)	4.92 ± 0.15a	4.64 ± 0.13b	4.22 ± 0.13c	3.72 ± 0.10d
Techno‐functional properties				
Water content (%)	7.68 ± 1.4a	7.57 ± 1.5a	8.46 ± 1.3a	8.46 ± 1.6a
Water solubility (%)	84.57 ± 4.5a	85.46 ± 5.0a	85.46 ± 6.0a	87.24 ± 4.7a
Water swelling (%)	151.33 ± 13a	154.00 ± 11a	157.56 ± 10a	160.23 ± 12a
Water activity (aw)	1.20 ± 0.30a	1.25 ± 0.25a	1.40 ± 0.23a	1.51 ± 0.27a
Hygroscopicity (%)	38.28 ± 2.5a	39.17 ± 2.0a	40.06 ± 2.8a	41.84 ± 2.3a
Hydrophobicity (μg/g)	6.50 ± 0.70a	7.12 ± 0.65a	7.57 ± 0.70a	8.01 ± 0.77a
Water‐holding capacity (g/g)	4.45 ± 0.40a	4.54 ± 0.36a	4.72 ± 0.44a	4.81 ± 0.46a
Oil‐holding capacity (g/g)	1.96 ± 0.22a	2.05 ± 0.20a	2.23 ± 0.35a	2.40 ± 0.27a
Emulsifying activity (%)	68.54 ± 4.0a	69.43 ± 4.3a	69.43 ± 4.0a	71.22 ± 4.5a
Emulsion stability (%)	57.86 ± 3.0a	57.86 ± 3.3a	56.08 ± 4.4a	56.08 ± 3.5a
Foaming capacity (%)	56.08 ± 4.3a	57.86 ± 4.0a	57.86 ± 4.7a	60.53 ± 5.5a
Foam stability (%)	41.84 ± 3.7a	40.95 ± 3.5a	43.62 ± 4.0a	45.40 ± 4.3a

*Note*: The values are expressed as means ± SD for three replicates. Mean values with different letters within a row are significantly different (*p* < .05).

### 3.7 Techno‐functional properties of gelatin‐protein hydrolysate particles

The techno‐functional parameters of gelatin materials were water content (7.68%), water solubility (84.57%), water swelling (151.33%), water activity (1.20), hygroscopicity (38.20%), hydrophobicity (6.50 μg/g), emulsification activity (68.54%), emulsification stability (57.86%), foam expansion activity (56.08%), foam stability (41.84%), water‐holding capacity (4.45 g/g), and oil‐holding capacity (1.96 g/g; Table 3). Techno‐functional determinations showed that gelatin showed low water content and hydrophobicity and high solubility, swelling, water activity, and hygroscopicity. Gelatin also exhibited high water‐holding capacity, oil‐holding capacity, emulsifying activity, and foaming activity. The techno‐functional parameters measured in this study were similar to those reported by Casanova et al. (2020) on saithe skin gelatin and Joy et al. (2023) on fish processing waste gelatin.

Adding protein hydrolysate causes minor but nonsignificant changes in water content (increase), water solubility (increase), water swelling (increase), water activity (increase), hygroscopicity (increase), hydrophobicity (increase), emulsification activity (increase), emulsification stability (decrease), foam expansion activity (increase), foam stability (increase), water‐holding capacity (increase), and oil‐holding capacity (increase) of powdered gelatin (Table 3). To our knowledge, there are no reports on the microencapsulation of *Satureja* protein hydrolysate in gelation or other protein and carbohydrate polymers. Obeidnejad et al. (2024) reported that incorporating *Satureja bakhtiarica* essential oil with a hydrophobic nature significantly changes gelation's water‐binding, oil‐holding, foaming, and emulsion capacities. Akbarbaglu et al. (2024) suggested that the incorporation of Arthrospira bioactive peptides within gum arabic, whey protein concentrate, pectin, and alginate results in an improvement of the physico‐chemical, techno‐functional, textural, and functional properties and stability of the bioactive protein. The same results were obtained by the incorporation of chlorella bioactive peptides within gum arabic, whey protein concentrate, pectin, and alginate by Gharehbeglou et al. (2024).

The techno‐functional properties of the protein depend on various internal protein characteristics like; primary structure (i.e. the amino acid sequence, amino acid composition, distribution of hydrophilic and hydrophobic amino acid, the position of cysteine amino acid), secondary structure (the length, sequence, and position of α‐helix, β‐sheet), tertiary structure (globular or fibrous protein), quaternary structure (heteromer or homomer), protein size, protein mass (molecular weight), protein net charge (isoelectric point), protein conformation, folding/unfolding status, surface hydrophobicity, amphipathic character, molecular flexibility and segmental mobility, and the existence of noncovalent interactions, including electrostatic, hydrophobic, and hydrogen bonding (Kumar et al., 2022). Furthermore, the techno‐functional activity of proteins depends on various external factors like protein concentration, solvent, temperature, ionic strength, salts (salting‐in and salting‐out), ingredients, protein treatment (germination, fermentation, soaking, toasting, and autoclaving), protein etching, protein oxidation, reducing agents, protein cross‐linking, non‐protein components, and mechanical stress (Yousefi & Abbasi, 2022). The gelatin structure is very cooperative, and the structural changes of gelatin under intrinsic and external factors will be less relevant. In our experiments, protein hydrolysates were incorporated into the gelatin matrix. The nonsignificant change in the protein hydrolysate's techno‐functional activity might be attributed to the same character of these components. Both protein (gelatin) and protein hydrolysate are composed of amino acids.

The water retention capacity of protein (water‐holding capacity, water content, water activity, hygroscopicity, wettability, swelling, water retention, solubility) is the capacity of the protein to retain water (physically entrapped water, hydrodynamic water, bound water, capillary water) in the polymer matrix. The volume of water associated with a protein is linked to its amino acids (in gelatin, proline and hydroxyproline), charged residues (acidic and basic amino acids), conformation (globular or fibrous), and hydrophobicity (hydrophobic amino acids). *Satureja* protein with high amounts of acidic and basic amino acids must improve the water‐binding capacity of gelatin, but this change is nonsignificant (Achouri et al., 2020).

The oil retention capacity of proteins depends on the existence of noncovalent bonds (Van der Waals, hydrogen bonds, hydrophobic, and electrostatic) that contribute to lipid‐to‐protein interactions. Oil retention capacity depends on the surface availability of nonpolar hydrophobic amino acid chains. As a result, protein denaturation or protein partial hydrolysis exposes hydrophobic regions and thus increases the oil‐holding capacity value. *Satureja* protein with high amounts of hydrophobic amino acids must improve the oil‐binding capacity of gelatin, but this change is nonsignificant (Brar et al., 2023).

Emulsion and foam formation are strongly influenced by protein surface properties. By reducing surface tension, emulsifiers or foaming agents facilitate the generation of stable oil–water and air–water borders. The functional surface activity of a protein depends on its conformational factors (distribution of hydrophilic and hydrophobic residues, flexibility, conformational stability, and segmental mobility) and external factors (temperature, pH, and ionic strength; Brar et al., 2023). *Satureja* protein with high amounts of hydrophobic, basic, and acidic amino acids improved the emulsion and foam capacity of gelatin.

## 4 CONCLUSION

*Satureja* protein hydrolysate may have various therapeutic effects due to the presence of valuable components of functional/essential/nonessential amino acids, suggesting this plant can be used as a spice, food additive, and food ingredient in the food and medical industries. *Satureja* protein hydrolysate demonstrated great anti‐amylase and anti‐lipase capacity. *Satureja* protein hydrolysate has potential uses in treating oxidative stress, diabetes, and other disorders. Nonetheless, further information must be provided regarding the molecular mechanisms of oxidative stress mitigation by *Satureja* peptides. Future studies should be conducted to investigate the role of *Satureja* protein hydrolysate in various food formulations as additives with potential economic value. Microencapsulation of protein hydrolysate to gelation dispersion causes minor changes in pH, electrical conductivity, osmolarity, surface tension, zeta potential, particle size, shear stress, and viscosity of the gelatin solution. Microencapsulation of protein hydrolysate to gelation causes minor, nonsignificant changes in techno‐functional properties like water content, water solubility, water swelling, hygroscopicity, hydrophobicity, emulsification activity, emulsification stability, foam expansion activity, foam stability, oil‐holding capacity, and water‐holding capacity. With excellent antioxidant capacity and anti‐amylase and anti‐lipase activity, *Satureja* protein hydrolysate microencapsulated in gelatin can be used as a carrier in nutritional products for the treatment of obesity, diabetes, and oxidative stress and as a natural preservative for the food industry.

## AUTHOR CONTRIBUTIONS

**Gholamreza Kavoosi:** Methodology; formal analysis; writing – original draft; writing – review and editing; visualization; funding acquisition. **Elham Obeidnejad:** Conceptualization; methodology; validation; writing – original draft; visualization; supervision. **Mohammad Jamal Saharkhiz:** Writing – review and editing. **Sayed Mohammad Shafiee:** Writing – review and editing.

## CONFLICT OF INTEREST STATEMENT

The authors declare no conflicts of interest. The sponsors had no role in the design of the study; in the collection, analyses, or interpretation of the data; in the writing of the manuscript; or in the decision to publish the results.

## Supporting information


Data S1.

## Data Availability

The corresponding author will deliver the information assisting the findings of this study upon reasonable request.
