# Supplementary material for: Chemical composition, functional properties, physico‐chemical properties, and techno‐functional characteristics of Satureja protein hydrolysate stabilized in a gelatin matrix
Source: Food Sci Nutr. 2024 Aug 20;12(10):8030–42. doi: 10.1002/fsn3.4344 (PMC11521751; doi:10.1002/fsn3.4344)
Supplement: Supplementary file 1 — Data S1. [file FSN3-12-8030-s001.doc]

***Chemical composition, nutritional quality, functional properties, physico-chemical properties, and techno-functional characteristics of Satureja protein stabilized in a gelatin matrix***

Elham Obeidnejad^1^, Gholamreza Kavoosi^1*^, Mohammad Jamal Saharkhiz^2^, Sayed Mohammad Shafiee^3^,

^1^Department of Biotechnology, School of Agriculture, Shiraz University, Shiraz, Iran.

^2^Department of Horticultural Science, School of Agriculture, Shiraz University, Shiraz, Iran.

^3^Department of Clinical Biochemistry, School of Medicine, Shiraz University of Medical Sciences, Shiraz, Iran.

*Corresponding author: Email: [ghkavoosi@shirazu.ac.ir](mailto:ghkavoosi@shirazu.ac.ir)

**Graphic abstract**


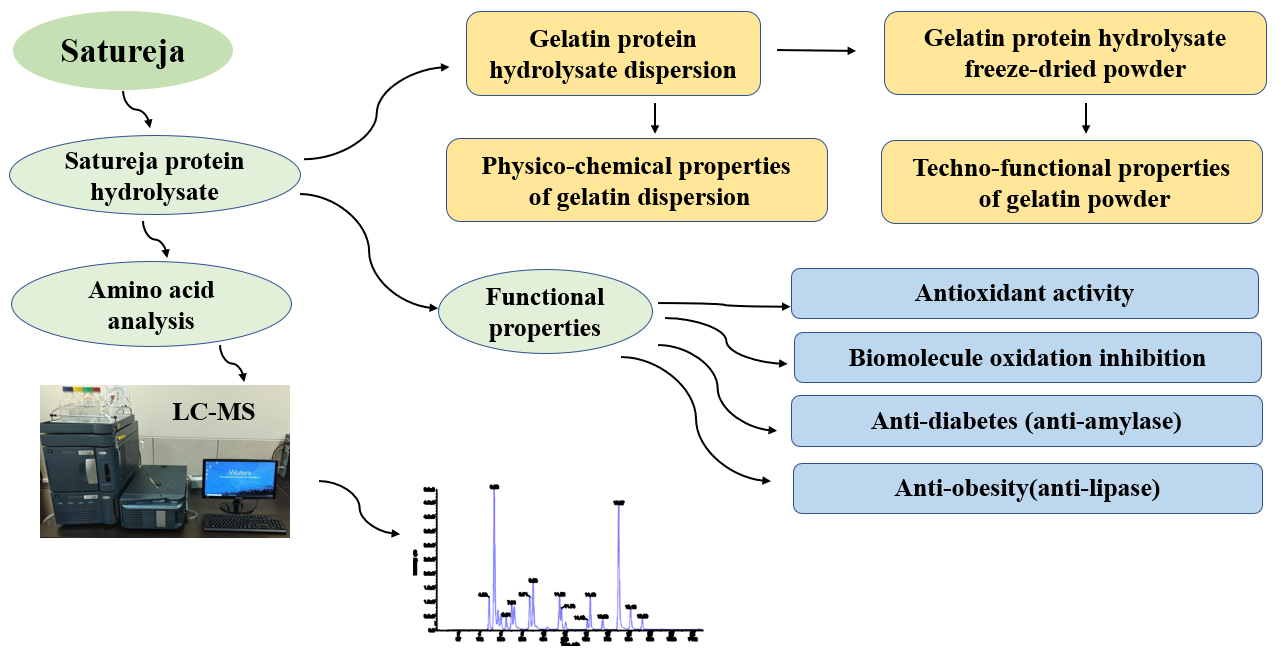


**Highlight**

1. Satureja had excellent essential/non-essential index and protein nutritional quality.

2. Satureja protein hydrolysate exhibits excellent antioxidant capacity.

3. Satureja protein hydrolysate inhibits amylase and lipase activities.

4. Satureja protein introduced as techno-functional ingredient for bioactive food products.

**2. Materials and methods**

**2.1. Herbal materials**

*Satureja hortensis* L. leaves were taken from an experimentation field. Following the identification of plant species, the voucher specimens (herbarium no. 538F19) were added to the herbarium at Shiraz University. Aerial parts of *Satureja sahendica* Bornm (Herbarium number: PH-1582) were prepared from Mazhin, Lorestan, and *Satureja rechingeri* Jamzad (Herbarium number: PH-1348) were collected from wild-growing plants at the full flowering stage in Dehloran, Ilam (western parts of Iran). *Satureja bakhtiarica* Bunge (Herbarium number MPH-1577) was also collected from Semirom, Isfahan (central part of Iran). Additionally, *Satureja khuzistanica* Jamzad (Herbarium number MPH-1588) was collected from Mazhin, Lorestan. *Satureja mutika* (Herbarium number MPH-1356) was prepared from Dehloran, Ilam (western districts of Iran) in the whole ﬂowering level (Taban et al., 2021). Professor Ahmad Reza Khosravi (Faculty of Science, Shiraz University, Shiraz, Iran), as an experienced botanical taxonomist, taxonomically recognized and confirmed this plant. The plant material was dried in the shade and converted into powder in a household mixer for later utilities.

**2.2. Amino acid extraction and profiling**

Before protein extraction, assafoetida fat, and essential oil were removed by mixing assafoetida with hexane (1:5 w/v) and stirring the mixture for one day at room temperature using a shaker at 150 rpm. The hexane phase (lipid and essential oil) was separated and defatted materials were dried at room temperature for 10 h. For protein extraction, defatted materials were mixed with 300 mM NaCl solution (1:5 w/v). For complete solubilization of protein and amino acids, pH was raised to 9-10 using NaOH. The upper phase was separated by centrifugation at 3000 g for 10 min. The supernatant pH declined to 7.0 with 500 mM HCl. The obtained protein suspension was powdered by freeze-drying and reserved in the refrigerator until experiments. For amino acid extraction, the assafoetida protein materials (1.0 g) were mixed with 10 mL of 6M HCl and incubated at 100°C for one day. The undigested components are separated by centrifugation at 3000 g for 10 min. The obtained amino acid suspension was powdered by freeze-drying and kept in a refrigerator until experiments. For amino acid profiling, the protein hydrolysate was dissolved in 500 mM NaCl solution (10 mg/mL). The chemical composition of protein hydrolysate was analyzed with liquid chromatography-tandem mass spectrometry (LC-MS/MS) (Agilent mass spectrometer, G1313) on an amino acid analyzer column. The amino acids separation was carried out on an amino acid analyzer column (C18 reversed-phase, length=100 mm, inner diameter=3 mm, pore size=100 Å, particle size= 2.7 µm) at a temperature of 40 °C with a 0.01 mL injection volume. The mobile solutions were A (0.2% formic acid in water) and B (0.2% formic acid in acetonitrile). The elution program was a linear gradient, commencing at 5.0% of solution B and increasing to 100% in 15 minutes. The flow rate of the mobile solution was 0.25 mL/min. The ion electrospray ionization (ESI) source obtained a mass spectrum in the m/z range of 30-3000.

The protein quality was evaluated by measuring the protein efficiency ratio, biological value, and the total profile of essential and non-essential (incl. aromatic, hydrophobic, sulfur, flavor, bitter, and sweet) amino acids (Table S1 is supplementary file).

| **Table S1. Classification and nutritional index of amino acids.** | |
| --- | --- |
| **Nutritional index** | **Calculation Formula** |
| Essential amino acid | histidine. isoleucine, leucine, lysine, methionine, phenylalanine, threonine, tryptophan, valine |
| Non-essential amino acid | alanine, arginine, asparagine, aspartic acid, cysteine, glutamic acid, glutamine, glycine, proline, serine, tyrosine |
| Ketogenic amino acid | leucine, lysine, isoleucine, phenylalanine, tryptophan, tyrosine |
| Glucogenic amino acid | histidine. methionine, threonine, valine, alanine, arginine, asparagine, aspartic acid, cysteine, glutamic acid, glutamine, glycine, proline, serine |
| Branched chain amino acid | leucine, isoleucine, valine |
| Flavor amino acid | aspartic acid, glutamic acid |
| Sweet amino acid | threonine, serine, glycine, alanine, proline |
| Bitter amino acid | valine, methionine, isoleucine, leucine, phenylalanine, histidine, arginine, tryptophan |
| Aromatic amino acid | phenylalanine, tyrosine, tryptophan |
| Sulfur amino acid | methionine, cysteine |
| Acidic amino acid | glutamic acid, aspartic acid |
| Basic amino acid | histidine, lysine, arginine |
| Hydrophobic amino acid | alanine, proline, cysteine, valine, methionine, phenylalanine, isoleucine, leucine |
| Protein efficiency ratio-1 | -0.684 + 0.456(leucine) - 0.047 (proline) |
| Protein efficiency ratio-2 | -468 + 0.454 (proline) - 0.105 (tyrosine) |
| Protein efficiency ratio-3 | -1.816 + 0.435(methionine) + 0.780 (leucine) + 0.211(histidine) - 0.944(tyrosine) |

**2.7. Total antioxidant capacity (ABTS)**

According to the literature (Heydari-Koochi et al., 2022; Siahbalaei et al., 2020), antioxidant capacity was assessed by mixing the different concentrations of assafoetida solution with ABTS stock solution and recording the light absorbance (734 nm). Trolox was used as standard control.  The total antioxidant capacity of essential oil was evaluated by mixing the essential oil (20 µL) with 1000 µL of 2,2-azino-bis (3-ethylbenzothiazoline 6-sulfonic acid) (ABTS) radical solution (7 mM ABTS and 2.54 mM potassium persulfate and one day in dark) and monitoring light absorbance at 734 nm. A calibration graph was drawn based on Trolox (10 mg/mL) as a standard reference. The antioxidant potential of essential oils was measured in milligrams of Trolox equivalents (TE) per gram of essential oil. The percentage of radical inhibition and the 50% inhibitory concentration (IC_50_) were calculated using the change in absorbance at 734 nm. All measurements were performed in triplicate.

**2.8. Glucose autoxidation inhibition assay**

Different amounts of amino acid samples (100, 200, 300, 400, 500 µg/ml) were incubated with a reaction solution containing sodium benzoate (1 mM), glucose (500 mM), and copper (II) sulfate (100 mM) at ambient temperature for four days. The fluorescence intensity of the solutions was read at 310 nm (excitation) and 410 nm (emission). The percentage reduction in fluorescence intensity was taken as the percentage inhibition in glucose oxidation as follows: percent inhibition = [(fluorescence intensity in the absence of amino acid - fluorescence intensity in the presence of amino acid)] / fluorescence intensity in the absence of amino acid × 100. Ethylenediaminetetraacetic acid (EDTA, 1.0 mM) was used as a positive control. Antioxidant / anti-diabetic activity index (AAI) was calculated as follow: AAI = IC_50_ of positive control / IC_50_ of amino acid sample. The following standard was used to qualify AAI values for amino acid extract: poor activity <0.05 <moderate activity <1.0 <strong activity <2.0 <very strong activity.

**2.9. Lipid peroxidation inhibition assay**

Different amounts of amino acid samples were incubated with 1000 µl of LDL solution (1.0 mg/mL). Instantly, 1000 µl of cupric sulfate (10 µM) was added and kept warm at ambient temperature for 10 h. The percent reduction of absorbance at 234 nm was taken as the percentage of lipid oxidation inhibition as follows: percent inhibition = [(Absorbance in the absence of amino acid - Absorbance in the presence of amino acid)] / Absorbance in the absence of amino acid × 100. Butylated hydroxytoluene (BHT, 10 mg/ml) was used as a control.

**2.10. Protein oxidation inhibition assay**

Different amounts of amino acid samples were incubated with 500 µl gelatin solution (1.0 mg/ml) and 500 µl of malondialdehyde solution (1.0 mg/ml) in a microplate. The microplate was incubated at ambient temperature for 20 h. Light absorbance was recorded at 245 nm. Butylated hydroxytoluene (BHT, 10 mg/ml) was used as control. The reduction in light absorbance at 245 nm was taken as the percentage inhibition in protein peroxide production as follows: percent inhibition = [(Absorbance in the absence of amino acid - Absorbance in the presence of amino acid)] / Absorbance in the absence of amino acid × 100.

**2.11. Protein glycation inhibition assay**

Different amounts of amino acid extracts were incubated with 500 µl gelatin solution (1.0 mg/ml) and 500 µl of glyceraldehyde solutions (40 mg/ml). The microplate was then kept warm at ambient temperature for 4 days. The fluorescence of solutions read at 370 nm (excitation); and 440 nm (emission). The percent reduction in fluorescence intensity was taken as the percentage inhibition in protein glycation as follows: percent inhibition = [(fluorescence intensity in the absence of amino acid - fluorescence intensity in the presence of amino acid)] / fluorescence intensity in the absence of amino acid × 100. Aminoguanidine (10 mg/ml) was used as a positive control

**2.12. Amylase inhibitory activity**

Following our previously reported procedure, the amylase activity in the absence and presence of inhibitors was determined by measuring the rate of starch breakdown using an iodine reagent and light absorbance recording at 580 nm (Heydari-Koochi et al., 2022; Siahbalaei et al., 2020). In Brief, the amylase solution (50 µL of 2.0 unit/mL) was pre-incubated with inhibitors at ambient temperature for 30 min. Then, the starch solution (100 µL of 2-10 mg/mL) was added to the reaction solution and incubated at ambient temperature for 30 minutes. Then 100 µL of iodine reagent (5 mM KI and 5 mM I_2_) was added to the solution to determine undigested starch. The absorbance (A) was recorded at 580 nm. A calibration graph was drawn based on acarbose as a positive control for amylase inhibition. The anti-amylase potential was measured in milligrams of acarbose equivalents (AE) per gram of samples. The percentage of amylase inhibition and the 50% inhibitory concentration (IC50) were determined according to absorbance changes. The Michaelis-Menten and Lineweaver-Burk plots were employed to determine the amylase kinetic parameters. Acarbose, essential oil, linalool, and limonene (300 µg/mL) were used as inhibitors, and starch (0-10 mg/ml) as substrate. The amylase activity (µg/min) was determined by measuring the rate of starch breakdown per minute for 30 minutes using light absorbance recording at 580 nm. Vmax and Km of amylase were determined using the Lineweaver-Burk plot (Siahbalaei *et al*., 2021). All measurements were performed in triplicate.

**2.13. Lipase inhibition**

Following our previously reported procedure the lipase activity in the absence and presence of inhibitors was determined by measuring the rate of p-nitrophenyl glucoside (pNPG) breakdown using light absorbance recording at 405 nm (Stefanucci et al., 2019). Orlistat was used as a positive control. Lipase (50 µL of 2.0 U/mL) was incubated with inhibitors (50 µL of 30-600 µg/mL) for 30 min. After the incubation, p-nitrophenyl butyrate solution (pNPB, 100 µL of 10 mg/mL) was added to the blend. The blends were incubated at ambient temperature for an extra 30 min. The light absorbance was measured at 405 nm. The anti-lipase activity was quantified as milligrams of orlistat equivalents in one gram of samples, with orlistat as a positive control for lipase inhibition. According to the change in absorbance, the percent of lipase inhibition and the inhibitory concentration leading to 50% inhibition (IC50) were measured. The Michaelis-Menten and Lineweaver-Burk plots were employed to determine the lipase kinetic parameters. Orlistat and bee product were used as inhibitors and pNPB (0-10 mg/mL) as substrate. The light absorbance monitoring at 405 nm was used to quantify pNPB breakdown per minute. Lipase activity (µg/min) was calculated and expressed as µg/min. The slope of light absorbance change versus time illustrated lipase activity. The Vmax and Km of lipase were calculated through the Lineweaver-Burk plot (Siahbalaei *et al*., 2021). All measurements were performed in triplicate.

**2.14. Physico-chemical and rheological properties**

The physico-chemical and rheological properties of diluted solution (1.0%) including; conductivity, osmolarity, zeta-potential, dynamic particle size, viscosity, and surface tension determined according to the practical approach at ambient temperature (Siahbalaei et al., 2020).

**2.14.1. Electrical conductivity:** To measure the conductivity, the Mettler-Toledo instrument (Cleantech, Schaffhausen, Switzerland) was utilized.

**2.14.2. Osmolarity:** The osmolarity of gelatin solution was measured by OSMOMAT 3000 osmometer (Gonotec, Germany) according to the comparison freezing point of water with gelatin solution.

**2.14.3. Particle size:** The hydrodynamic diameter of particles (particle size) and polydispersity were measured according to the principle of Dynamic Light Scattering (DLS) using the Horiba SZ-100 particle size analyzer (Japan).

**2.14.4. Zeta-potential:** The electrophoretic mobility and zeta-potential of gelatin particles were measured using the Phase Analysis Light Scattering (PALS) technique using the Horiba SZ-100 zeta-potential analyzer (Japan).

**2.14.5. Surface tension:** The surface tension was measured with a Du Nouy tensiometer (Kruss, Germany).

**2.14.6. Viscosity:** The quantitative viscosity was measured with the MCR302 rheometer (Anton Paar) at different shear rates (Siahbalaei et al., 2020).

**2.15. Techno-functional characterization**

Before experiments, the samples were powdered by freeze-drying. The techno-functional properties of powdered materials including; water content, water solubility, water activity, hygroscopicity, surface hydrophobicity, emulsification activity, emulsification stability, foam expansion activity, foam stability, oil-holding capacity, and water-holding capacity were measured as previously described (Bar et al., 2023; Kim, & Shin, 2022).

**2.15.1. Moisture Content:** The powdered sample (1.0 g) was placed in an air-drier oven for 2 hours at a temperature of 100°C. Finally, the samples were taken from the oven, cooled in desiccators, and weighed. The moisture content was calculated by the percentage of weight difference between samples before and after oven drying.

Water content (%) = 100 [(initial weight of the sample-weight of the dried samples)/initial weight of the sample)]

**2.15.2. Water solubility:** The powdered sample (1.0 g) was mixed with 20 mL of distilled water for 10 min at room temperature. The resulting solution was centrifuged at 4500 rpm for 10 min. After centrifugation, the supernatant (5 mL) was dried in an oven at 100°C until a constant weight was achieved. The mass of the sample obtained after drying was used to determine the solubility using the following equation:

Water solubility (%) = 100 × [(initial weight of sample-weight of dried supernatant)/initial weight of sample)]

**2.15.3. Swelling capacity:** The powdered sample (1.0 g) was homogenized with 10 mL of distilled water for 1 min, using a graduated tube, and kept at 25 °C for 1.0 h. The initial and final weight of the samples was measured, and the increase in sample weight after incubation indicates swelling capacity. The swelling capacity was calculated using the following equation:

Water sweeling (%) = 100 [(weight of the swelled sample)/initial weight of sample)]

**2.15.4. Hygroscopicity:** The powdered sample (1.0 g) was placed in a desiccator containing saturated NaCl solution (relative humidity 75%). Samples were kept at ambient temperature for five days. The initial and final weight of the samples was measured, and the increase in sample weight after incubation indicates hygroscopicity capacity. The hygroscopicity of powders was determined by the following equation:

Hygroscopicity (%) = 100× [(final weight of sample)/initial weight of sample)]

**2.15.5. Water Activity:** Water activity was determined using an Aqualab analyzer at 25°C after stabilization of the samples at this temperature for 40 min.

**2.15.6. Surface hydrophobicity:** The surface hydrophobicity capacity was evaluated based on the hydrophobic interactions between basic and aromatic amino acid residues of proteins and the anionic form of Coomassie Brilliant Blue G-250 which forms a complex that can be measured at 585 nm. Briefly, 1.0 mL of a solution of 5 mg/mL samples in ultra-pure water was mixed with 300 μl of a 0.1 mg/mL solution of Coomassie Blue in ultra-pure water. A control was prepared by mixing 1.2 mL of the phosphate buffer with 300 μl of the Coomassie Blue solution. Samples were stirred at 2000 rpm for 3 min and after, centrifuged at 2000g for 10 min at 4 °C. The supernatant was taken and centrifuged again under the same conditions. Finally, the light absorbance of the supernatant was measured at 585 nm. The amount of Coomassie Blue bound was calculated using the following formula:

Coomassie Blue bound (µg) =30 × [(A control – A sample)/ A control]

**2.15.7. Water-holding capacity:** The powdered sample (0.5 g) was homogenized with 5.0 mL of distilled water for 5 min, and kept at 25°C for 30 min. Subsequently, the test tubes were centrifuged at 15,000 x g for 20 min at 4°C. The supernatant was decanted, and the test tubes with the sediments were weighed. The Water-holding capacity was calculated as follows:

Water-holding capacity (%) = [(Final weigh of the tube with the sample – Initial weigh of the tube with the sample) / (Weigh of the sample)] × 100.

The water absorbed was converted in weight (g) by multiplying the respective density. The density of water was assumed to be 1.0 g/ml.

**2.15.8. Oil-holding capacity:** The powdered sample (0.5 g) was mixed with 5 mL of commercial corn oil in a pre-weighed centrifuge tube. The suspension was held at 25°C for 30 min at 3000 g. The tube was inverted for 25 min after decanting the separated oil layer to drain the excess before weighing. The supernatant was decanted, and the test tubes with the sediments were weighed. The oil-holding capacity (%) was calculated as follows:

Oil-holding capacity (%) =100 × [(Final weigh of the tube with the sample – Initial weigh of the tube with the sample) / (Weigh of the sample)]

The oil absorbed was converted in weight (g) by multiplying the respective density. The density of oil was determined to be 0.88 g/ml.

**2.15.9. Emulsion activity and stability:** The powdered sample (1.0 g) was taken and mixed with 1- mL of distilled water, and 10 mL of commercial corn oil was added slowly and mixed after thorough dispersion. It was then centrifuged at 3000 rpm for 5 min, and the volume of oil separated from the sample was recorded. The ratio of the height of the emulsion to the total height was considered as the emulsion activity (%). The emulsion stability of the samples was determined by heating the fully prepared emulsion at 80°C for 30 min and then kept in cold water for 15 min. The emulsion was then centrifuged at 1300 g for 5 min, and the emulsion stability was determined by:

Emulsion activity (%) = 100 ×[(height of the emulsified layer / height of the whole layer in the tube)]

Emulsion stability (%) = 100 × (height of the emulsified layer remained after heating/height of the whole layer in the tube)

**2.15.10. Foaming capacity and stability:** The powdered sample (1.0 g) was whipped for 5 min with 20 mL of distilled water in a Waring blender. It was then poured into a 100 mL measuring cylinder. The foaming capacity was calculated as:

Foaming capacity (%) = 100 × [foam volume immediately after mixing/ starting volume of the liquid phase]

Foaming stability was calculated as the change in the volume of the foam after 1.0 h of mixing.

Foaming stability (%) = 100 × [foam volume after 1.0 h of mixing/ foam volume immediately after mixing]

**2.15.11. Gelling capacity (GC):** The samples were homogenized with distilled water to obtain a suspension at 10 % (w/v). The test tubes containing these suspensions were then heated for 1 hr in a boiling water bath followed by rapid cooling under cold tap water. The test tubes were then further cooled for 2 hr at 4°C. The lowest gelation concentration was when the sample from the inverted test tube did not fall or slip.


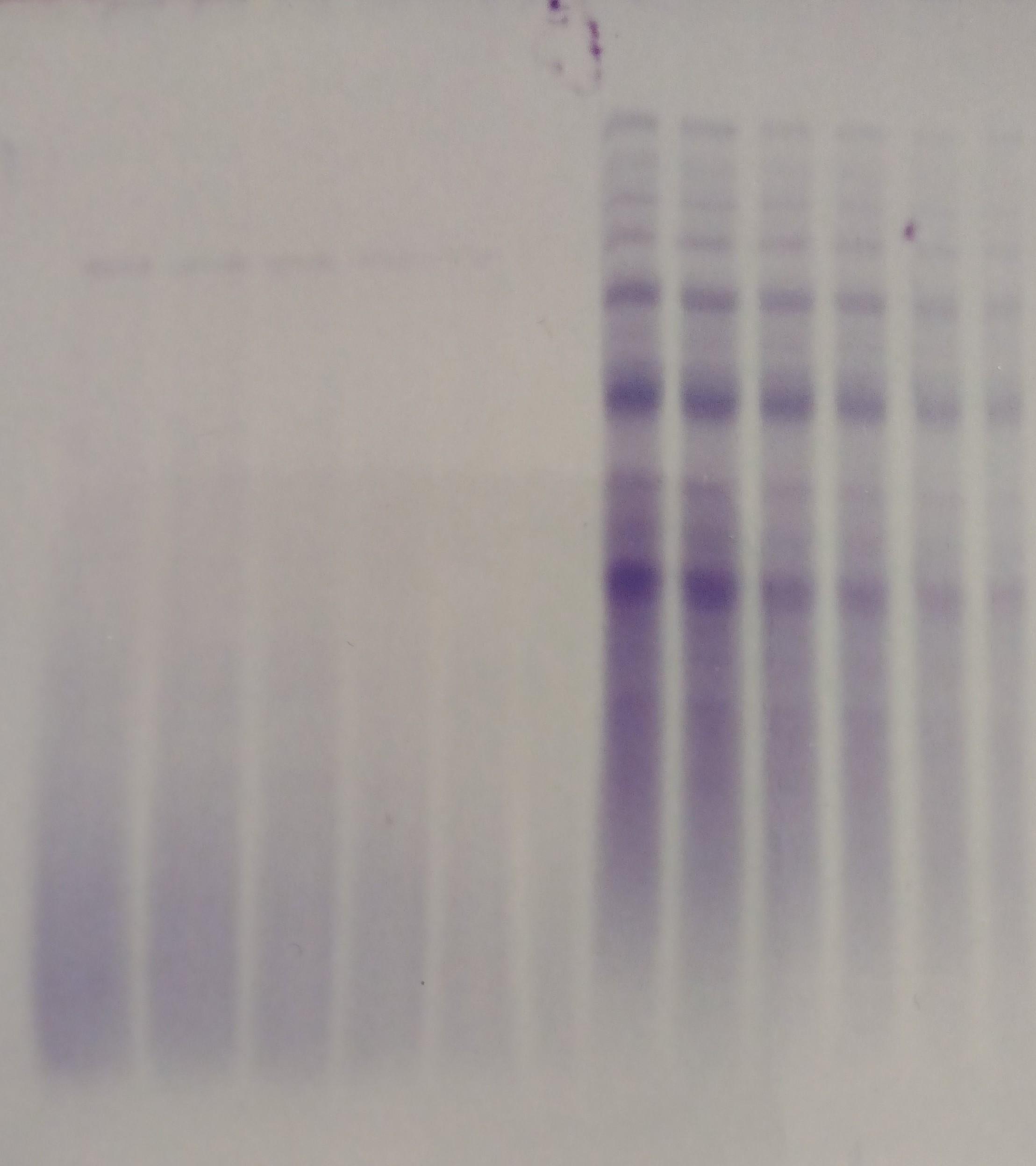


Figure S1. SDS-PAGE of Satureja protein hydrolysed by mild acid.


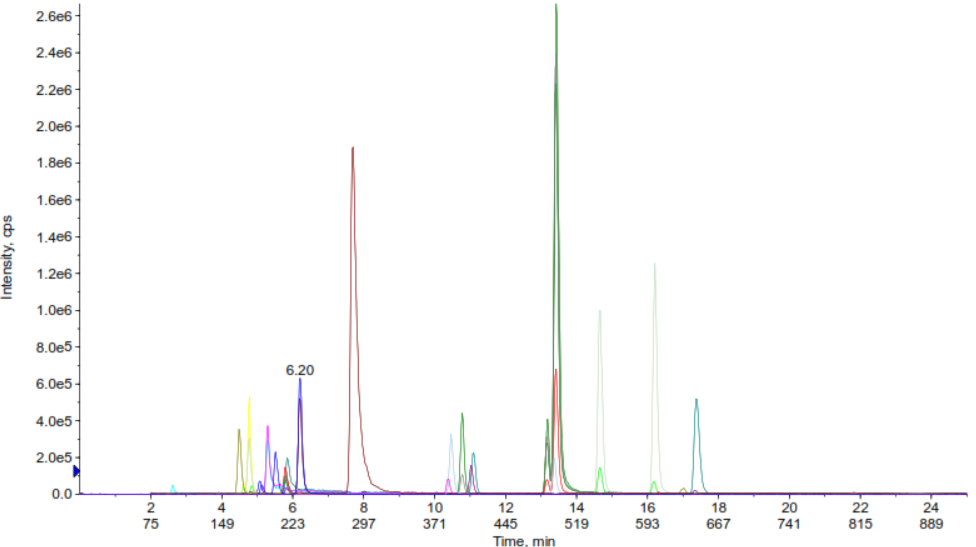


Figure S2. Total ion chromatogram of amino acid from satureja bakhtiarica.


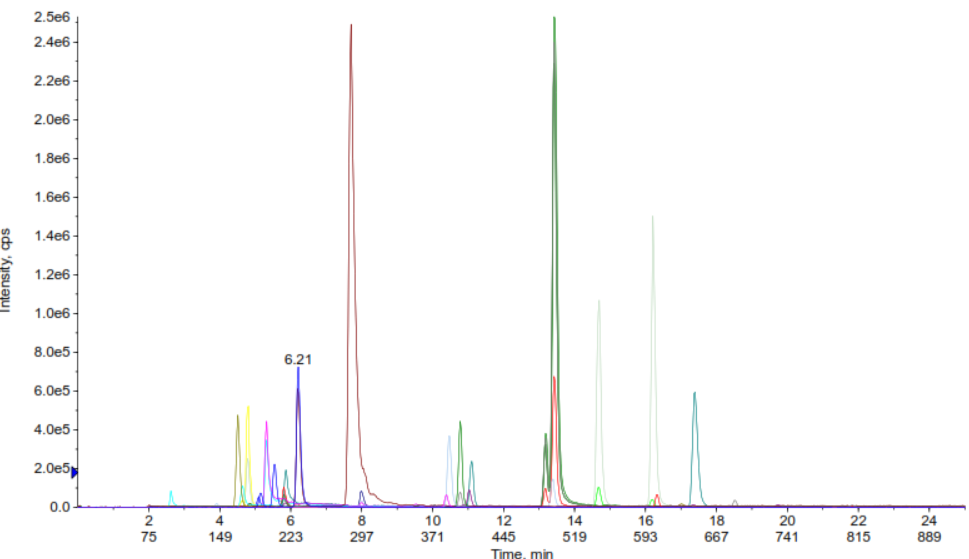


Figure S3. Total ion chromatogram of amino acid from satureja hortensis.


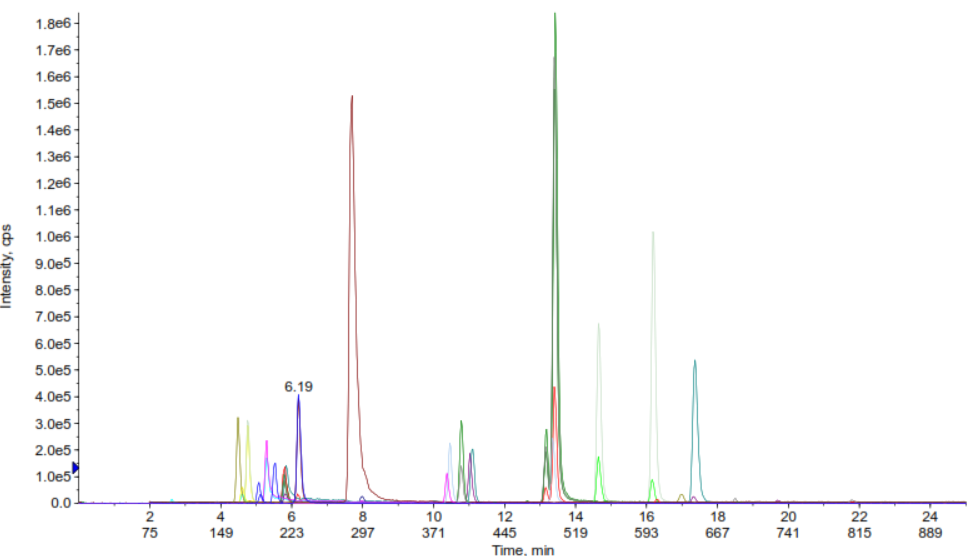


Figure S4. Total ion chromatogram of amino acid from satureja khozestanica.


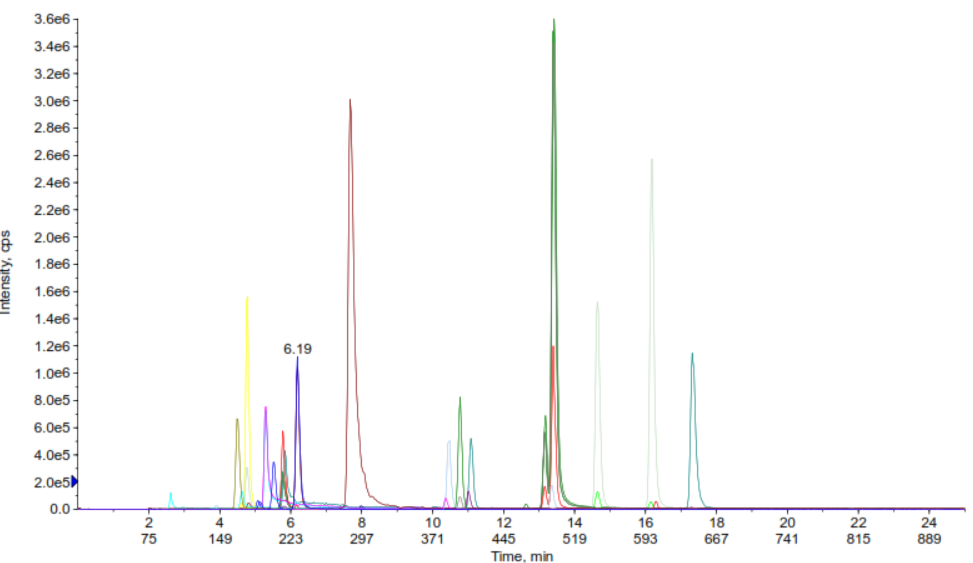


Figure S5. Total ion chromatogram of amino acid from satureja mutika.


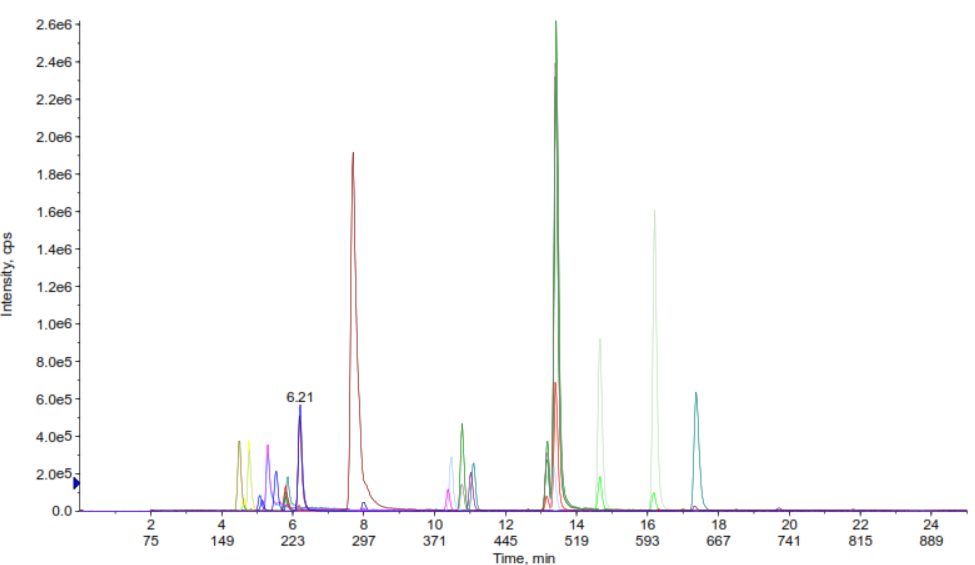


Figure S6. Total ion chromatogram of amino acid from satureja rechengeri.


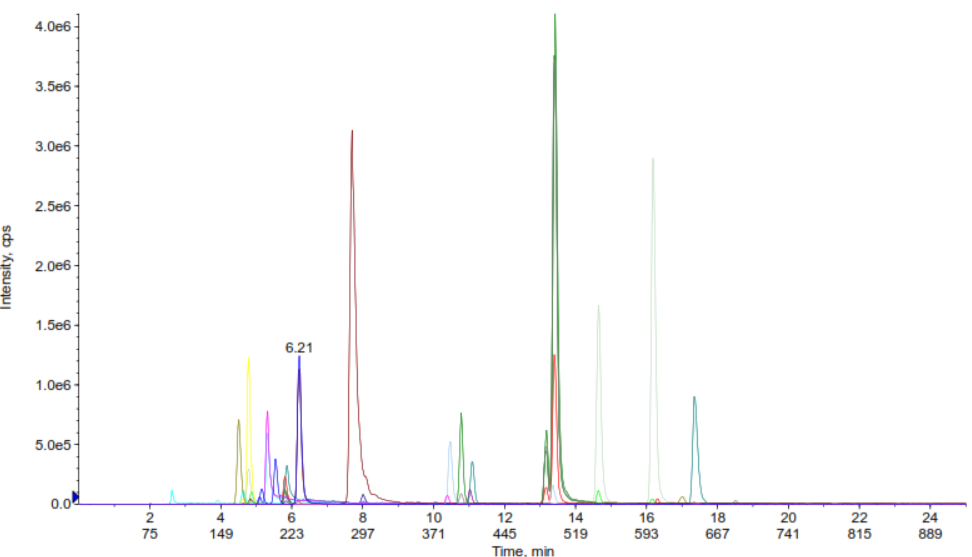


Figure S7. Total ion chromatogram of amino acid from Satureja sahendica.


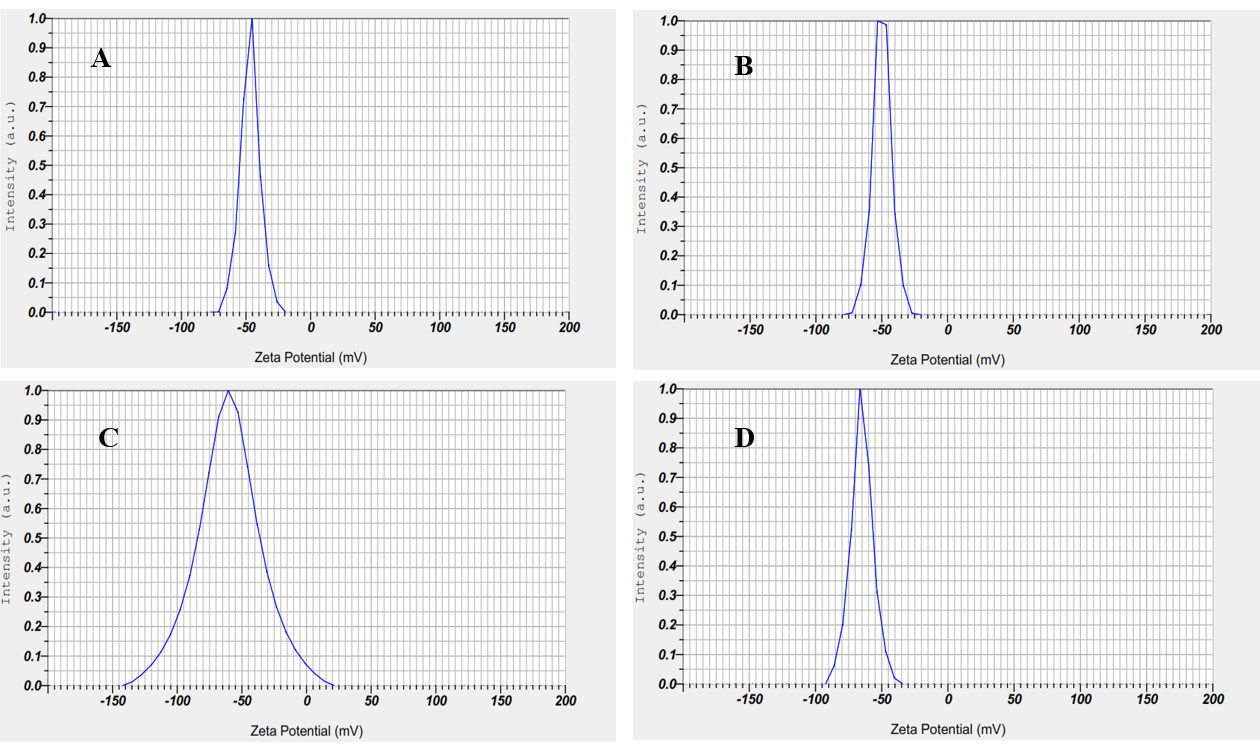


Figure S8. Zeta-potential profile of gelatin solution incorporated with Satureja protein hydrolysate.


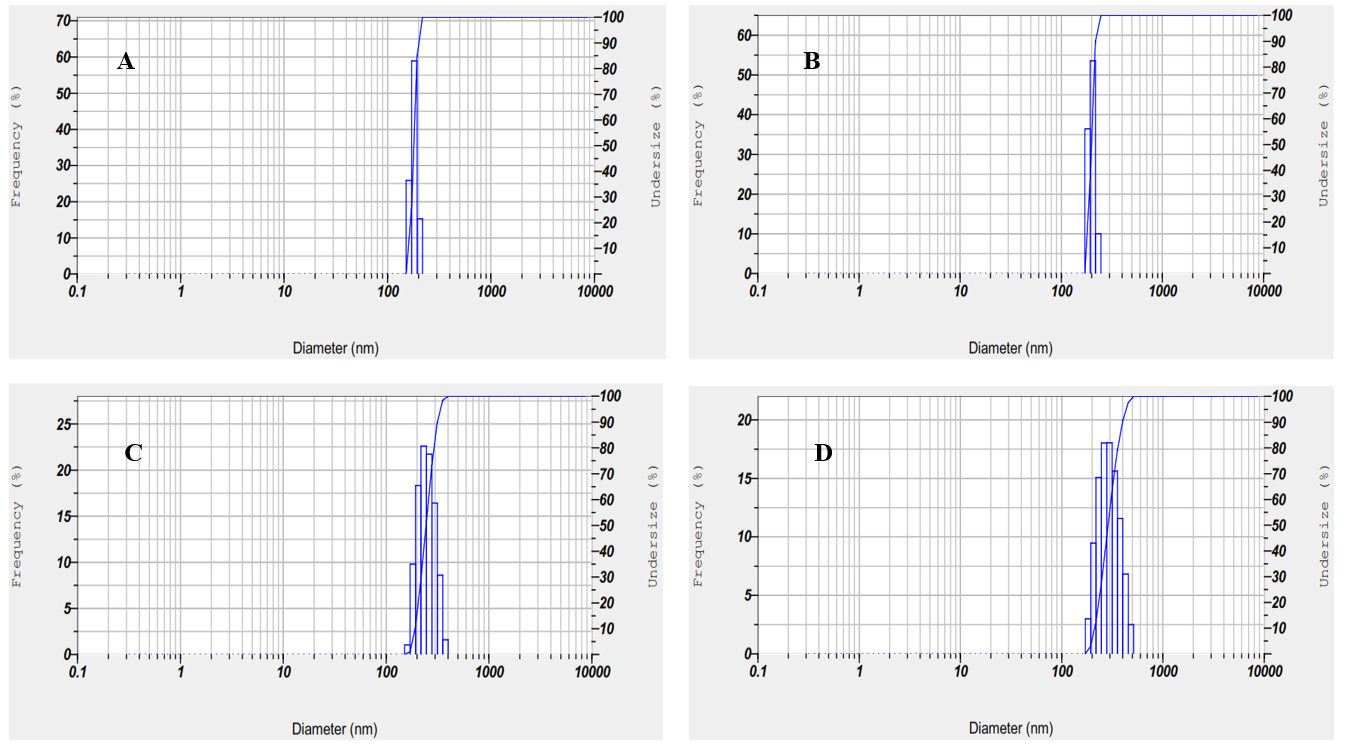


Figure S9. Particle size profile of gelatin solution incorporated with Satureja protein hydrolysate.


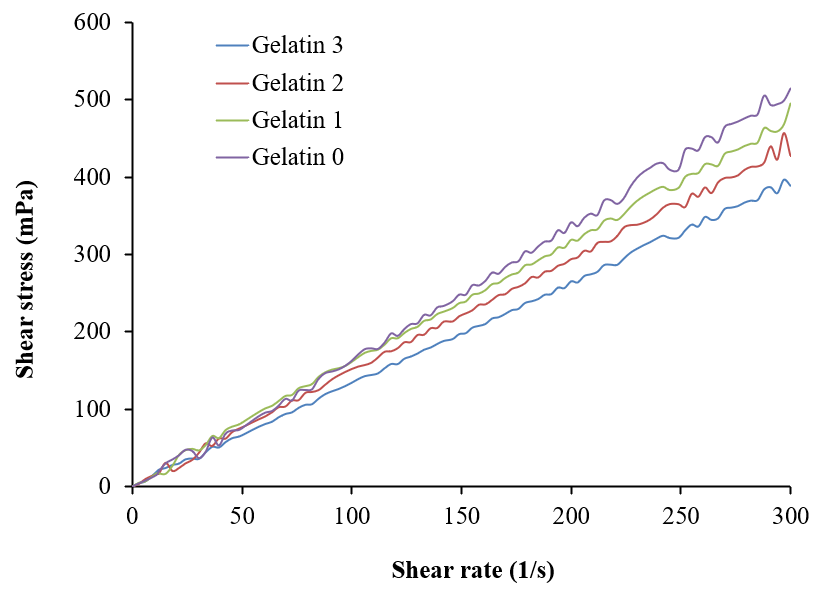


Figure S10. Shear stress-shear rate profile of gelatin solution incorporated with Satureja protein hydrolysate.


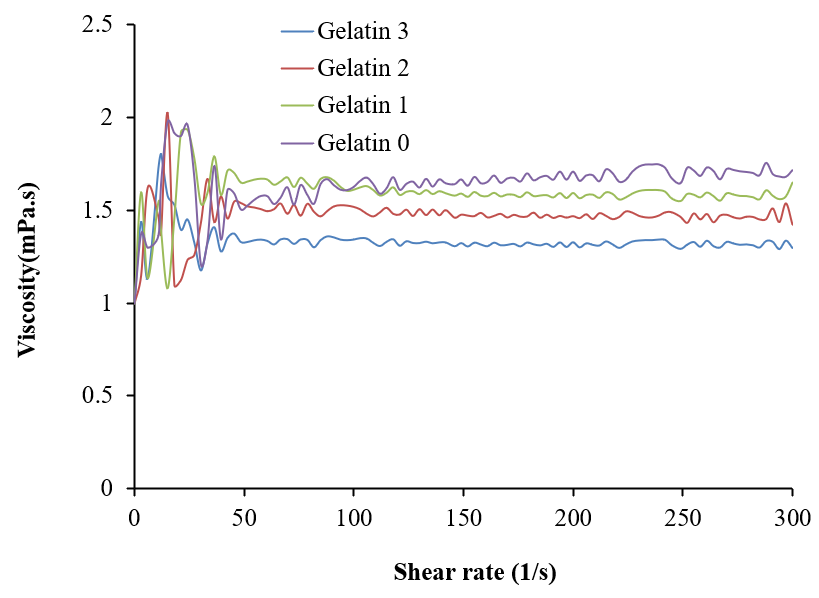


Figure S11. Viscosity -shear rate profile of gelatin solution incorporated with Satureja protein hydrolysate.
